# Pulsed Electric Field Treatment Enhances the Cytotoxicity of Plasma-Activated Liquids in a Three-Dimensional Human Colorectal Cancer Cell Model

**DOI:** 10.1038/s41598-019-44087-5

**Published:** 2019-05-20

**Authors:** Elena Griseti, Jelena Kolosnjaj-Tabi, Laure Gibot, Isabelle Fourquaux, Marie-Pierre Rols, Mohammed Yousfi, Nofel Merbahi, Muriel Golzio

**Affiliations:** 1 CNRS UMR 5089, Institut de Pharmacologie et de Biologie Structurale, IPBS, 205 Route de Narbonne, 31077 Toulouse, France; 2 Université Toulouse III- Paul Sabatier, CNRS UMR 5213, Laboratoire des Plasmas et Conversion d’Énergie, LAPLACE, 118 Route de Narbonne-Bât 3R3-31062, Toulouse, France; 3 Centre de Microscopie Électronique Appliquée à la Biologie, CMEAB, 133 route de Narbonne Rangueil, 31062 Toulouse, Cedex France

**Keywords:** Cell delivery, Permeation and transport

## Abstract

Cold atmospheric plasma and more recently, plasma-activated liquids (culture media, water or buffered solutions previously exposed to plasma), are gathering momentum in cancer cells treatment. Nevertheless, *in vitro* tests show that this novel approach is sometimes less efficient than expected. We here evaluate the mechanisms of action of the plasma-activated PBS and suggest to use electropermeabilization (EP) in combination with the plasma-activated phosphate-buffered saline (PBS), in order to potentiate the cytotoxic effect of the plasma activated liquid. Human multicellular tumor spheroids (MCTS), a three-dimensional cell model, which resembles small avascular tumors, was used to define the optimal treatment conditions for single and dual-mode treatments. MCTS growth, viability, and global morphological changes were assessed by live cell video-microscopy. In addition, the induction of caspases activation, the appearance of DNA damages, and cell membrane permeabilization, as well as the early modifications in the cellular ultrastructure, were examined by immunofluorescence, propidium iodide staining, confocal fluorescence microscopy and transmission electron microscopy, respectively. Altogether, our results show that a combined treatment resulted in an earlier onset of DNA damage and caspases activation, which completely abolished MCTS growth. This report is a proof of concept study evidencing that electropermeabilization greatly potentiates the cytotoxic effect of plasma-activated PBS *in vitro* in a three-dimensional cancer cell model.

## Introduction

Cold atmospheric plasma (CAP), a weakly ionized gas composed of reactive species, charged particles and photons, has been suggested in the last decade as a new modality for several biological and medical applications such as dentistry, sterilization, wound healing, tissue regeneration, and cancer treatment^1^. In the latter domain, CAP has demonstrated a potential capacity to inhibit tumor cells *in vitro* and *in vivo* in some types of cancer^2^. The cytotoxicity of CAP is mainly related to the reactive oxygen and nitrogen species (RONS), which are formed within the gas phase due to interactions between plasma and ambient air^3^ or in contact with treated surface^4^. RONS are present in plasma at relatively high concentrations, and include long-lived reactive species such as hydrogen peroxide and nitrite-nitrate anions^4^. Interestingly, studies suggest that plasma can specifically kill cancer cells without affecting normal cells^5,6^. In the field of cancer treatment, two approaches are proposed. The first approach consists of a direct treatment of cancer cells with the CAP, where the gaseous plasma species directly act on the cancerous cells. The second strategy, which is much more recent, involves an indirect treatment with the plasma-activated liquids (PALs)^7^, where liquids are at first exposed to CAP (or, in other words, are activated by the plasma), and are subsequently placed in contact with cancer cells. Such liquids include either cell culture media (PAM), or water (PAW) or physiological solutions such as phosphate buffered saline (PBS) or the NaCl saline solution (P-A PBS/NaCl, respectively)^8^. The use of PALs for cancer treatment is currently at an early stage of development, and the effects of PALs on cells are poorly understood. Interestingly, in addition to being cytotoxic to cancer cells, studies show that the cytotoxicity of plasma-activated solutions may be preserved over extended storage periods at −80 °C and even 4 °C^9^, meaning that stock supplies could be prepared in advance and used later, which would offer extensive advantages for biomedical applications. Another benefit of PALs in respect to direct treatments with plasma jets, is that liquids could be injected into deep-seated tumors, whereas direct CAP treatments are limited to surface applications. Data gathered from *in vitro* studies suggest PAM induces DNA damages and stops the proliferation of human colorectal cancer cells^10^. To date, our team has investigated the effect of PAM on several tumor types in a 3D cancer cells model, the multicellular tumor spheroid (MCTS). We previously showed that the response to the treatment is cancer cell type-dependent. Intriguingly, in FaDu head and neck cancer cells MCTS, a proliferation boost was observed^11^. On the contrary, cytotoxic effects were observed at the external layer of the MCTS, made of human colorectal cancer cells (HCT 116)^9^. Finally, the cytotoxicity of P-A PBS was reported for different cancer cell types including human glioblastoma^12,13^ and human pancreas adenocarcinoma cells^13^. These promising preliminary results suggest that PALs penetration might be limited, particularly when cells have tighter junctions, or when larger volumes of cells have to be treated.

With the aim to enhance RONS penetration, and thus to increase the efficiency of P-A PBS, we here suggest the use of the P-A PBS in combination with electropermeabilization (EP). The EP, which was first described in 1972 by Neumann *et al*. on vesicular membranes^14^, relies on the capacity of pulsed electric fields to modulate the electric transmembrane potential of cells, resulting in the generation of transient and localized permeant structures in cell membranes, while not altering the cellular homeostasis^15^. At well-defined electric pulses parameters (field strength, pulse duration, number of pulses), the increased permeability is transient and the viability of the cells is well preserved^16^. This capacity of pulsed electric field to reversibly permeabilize cell membranes is nowadays used to increase intracellular delivery of therapeutic molecules to cells and tissues. This approach thus enables the administration of lower local or systemic drug doses^17,18,19^. In the case of electrochemotherapy (ECT), which is a combined approach using chemotherapeutics and electropermeabilization, a fast and efficient transfer of anti-cancer drugs is enabled across transient membrane alterations, generated by EP. Protocols in the framework of the European Standard Operating Procedure on Electrochemotherapy (ESOPE protocol) standardized the clinical application of ECT^20^. In 2006, a prospective non-randomized study reported a complete 73.7% response rate for local tumor nodules treatment with ECT^21^. Ten years later, a prospective, observational study enrolling 376 patients with superficial metastases, who were subjected to ECT in 10 different research centers confirmed these results^22^. Electrogenotherapy (EGT) is also used in clinics and allows efficient electro-delivery of small RNA (siRNA and anti RNA), as the electric field reportedly acts on both the membrane permeabilization and the electrophoretic drag of the negatively charged small RNA molecules, and both processes are required to promote the translocation and traffic to the nucleus and finally contribute to gene expression^23,24,25^. Currently, new electrochemotherapy strategies are under development to extend ECT applicability to tumors with different histological hallmarks. Calcium electroporation was recently proposed as a simple and inexpensive tool for anticancer therapy^26,27^, and relies on the combination of EP with supraphysiological doses of calcium. This therapeutic approach supports the theory that other compounds, which are not necessarily complex chemical drugs, could be combined with electroporation to cure cancer.

In a similar context, a recent study investigated the effect of a sequential treatment of cells with EP and PAM, where the effect was compared in tumorigenic versus normal rat liver adherent monolayers of epithelial cells^28^, and demonstrated that combined treatment led to a better killing of tumor cells, while non-tumorigenic cells were less affected.

In order to test the efficacy of a treatment, three-dimensional (3D) cell culture model, the MCTS, can be used. The MCTS has particular interest in biomedical studies, especially in screening tests for cancer treatments, due to cellular 3D organization that mimics tumor tissues^29,30^. In this regard MCTS might include a proliferative, a quiescent and a necrotic layer, cell-to-cell communications channels, including gap junctions, desmosomes and electrical coupling, and its own extracellular matrix^31,32,33^. Thus, MCTS allows a better preclinical evaluation of anticancer therapeutic strategies and enables the prediction of the *in vivo* response. The 3D organization makes cellular spheroids particularly attractive for the evaluation of drug delivery efficiency upon EP^34^.

In this context, the aim of the present study was to evaluate the effect of P-A PBS in a 3D cancer cells model, and to potentiate the cytotoxic effect, by combining P-A PBS treatment with EP. The response to single and dual treatments was investigated within MCTS made of human colorectal cancer cells HCT 116, expressing the green fluorescent protein (GFP). The P-A PBS’s efficiency was monitored: (i) by live cells fluorescence microscopy, which allowed assessing macroscopic morphological changes and cell membrane permeability occurring in HCT 116-GFP MCTS over time; (ii) by transmission electron microscopy (TEM), which enabled us to evaluate modifications in MCTS ultrastructure; (iii) by immunofluorescence, after the immunostaining of phosphorylated histone H2A variant H2AX, which allows assessing DNA damages, and (iv) by a luminescent assay, determining the pro-apoptotic potential of the treatment, measuring caspase-3/7 activities in MCTS.

## Results

### Plasma exposure time-dependent cytotoxicity of P-A PBS in HCT 116-GFP MCTS

Prior the investigation of the effect of a dual treatment with P-A PBS and EP, we first selected the optimal conditions for PBS activation with the CAP jet. MCTS were treated with PBS activated with the plasma jet for 60, 120 or 240 seconds. Control MCTS were incubated for 4 hours either in PBS or in PBS exposed to the helium gas flow (no plasma) to obtain a control hyoertonic solution. The exposure to hypertonic solution did not affect MCTS growth, as their growth rate was similar to the MCTS incubated in cell culture medium (Supplementary Data, Fig. 1).

**Figure 1:**
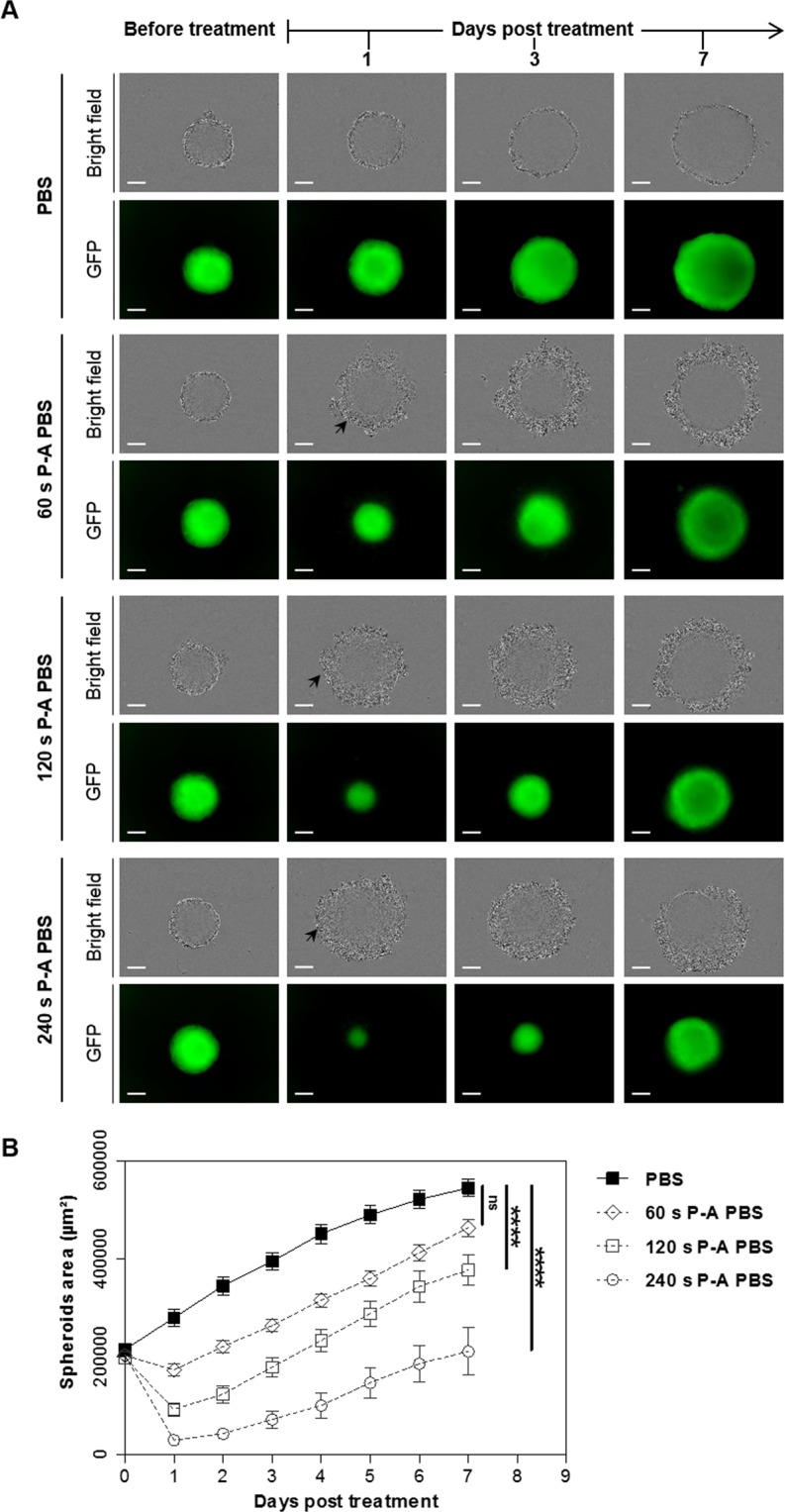
MCTS growth inhibition depends on the time of PBS activation with the plasma jet. HCT 116-GFP MCTS were incubated 4 hours in P-A PBS activated with the plasma jet for 60, 120 or 240 seconds. After treatment, MCTS were placed in culture medium for a growth follow-up during 7 days. (**A**) Bright field and GFP fluorescence micrographs of MCTS before treatment and at day 1, 3 and 7 post treatment. Black arrows indicate detached dead cells. Scale bar: 100 µm. (**B**) Growth curves plotted from GFP fluorescence area, mean ± standard error mean. N = 2 experiments, n = 6 MCTS per experiment per condition. Two-way ANOVA ****p < 0.0001.

The macroscopic appearance and the viability (which was correlated with the GFP fluorescence) of MCTS were daily monitored with a wide field microscope (Fig. 1). One day after the treatment, bright field micrographs evidenced detached cells (indicated by black arrows in Fig. 1A) in the periphery of the treated MCTS. Detached cells did not exhibit any green fluorescence. The width of the concentric rim of dead cells was the greatest in MCTS, which were treated with PBS activated with the plasma jet during the longest period. The growth curve (Fig. 1B) shows that MCTS lost 15, 55 and 85% of their area, respectively, at day 1 after the treatment with PBS, which was activated with a plasma jet for 60, 120 and 240 seconds, respectively. When PBS was activated for 60 or 120 seconds, MCTS were able to grow with a similar rate to controls. On the contrary, MCTS treated with P-A PBS, which underwent a 240 seconds activation, exhibited a slower growth rate. In a seven days follow up, MCTS were 84, 70 and 38% smaller than control, respectively, for PBS activated with a plasma jet for 60, 120 and 240 seconds.

Hydrogen peroxide H_2_O_2_ and nitrite NO_2_^−^ content were also quantified, and the osmolarity of P-A PBS was measured for different plasma exposure times (Supplementary Data, Table 1). The concentrations of the two species directly correlated with plasma exposure times and correlated with the trend observed in growth curves. PBS plasma jet exposure caused an increase in the osmolarity of the solution, which was proportional to the plasma exposure time, and was due to water evaporation.

Altogether, these results demonstrate that the cytotoxicity of P-A PBS depends on the time of exposure of the PBS to the plasma jet. Sixty seconds of exposure were not enough to obtain significant difference in MCTS growth seven days after treatment, compared to 120- and 240-seconds activation, where irreversible damages and a slower growth rate of MCTS were observed. While P-A PBS activated for 120 or 240 seconds both had a cytotoxic effect, the exposure of PBS to a plasma jet for 240 seconds highly impacted the osmolarity of the solution. As the osmolarity of the PBS activated for 120 seconds was in a tolerable range for the cells, we considered it as the most appropriate for the combination with EP. Moreover, the concentration of hydrogen peroxide within this solution was high enough to induce cytotoxicity to cancer cells, while being tolerated by normal cells^7,35^.

### Optimization of EP parameters for non-lethal and efficient MCTS reversible permeabilization

**Figure 2:**
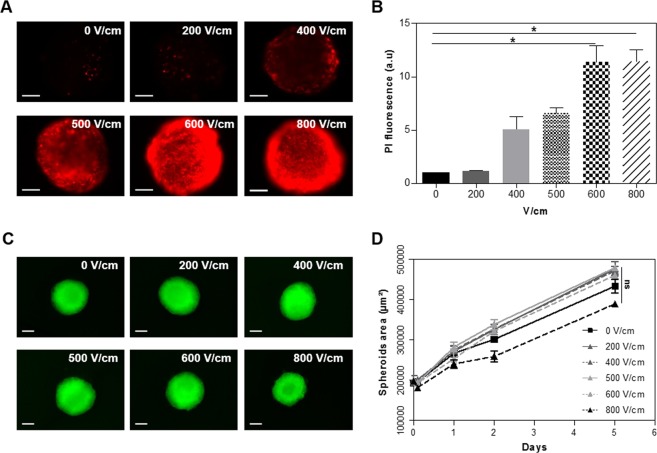
EP conditions optimization for MCTS reversible electropermeabilization. HCT 116-GFP MCTS were submitted to different electric field strengths (0, 200, 400, 500, 600, 800 V/cm) in PBS before being incubated (i) with 100 µM propidium iodide (PI) to assess the permeabilization or (ii) in culture medium for the growth follow up. (**A**) Wide field fluorescence micrographs (PI staining in red) obtained immediately after electropermeabilization. Scale bar: 100 µm. (**B**) Quantification of PI fluorescence according to EP condition. One-way ANOVA *p = 0.01 (**C**) GFP fluorescence micrographs 2 days after treatment. Scale bar: 200 µm. (**D**) MCTS growth curves over 5 days post treatment (determined from the area of GFP fluorescence). Two-way ANOVA showed no significant (ns) differences in MCTS growth over time. B and D displayed mean ± standard error mean; N = 2 experiments, n = 6 MCTS per experiment per condition.

In order to potentiate the cytotoxic effect of P-A PBS without harming the cells with a pulsed electric field, we defined the optimal conditions for a transient electropermeabilization. MCTS were submitted to EP according to parameters used in electrochemotherapy ESOPE protocols (eight pulses lasting 100 µs at a frequency of 1 Hz)^21^. Different electric field intensities were tested, from 200 to 800 V/cm, in order to select the intensity allowing efficient and reversible cell permeabilization, without affecting cell growth and viability. Permeabilization was evaluated on live MCTS shortly after EP with wide field fluorescent microscopy (Fig. 2A) using the propidium iodide (PI), a non-permeant red fluorescent DNA intercalating agent that penetrates only in cells exhibiting defaults in plasma membrane integrity. PI fluorescence was quantified (Fig. 2B). In parallel, MCTS were electropulsed and directly placed in culture medium, in order to evaluate the impact of EP on MCTS growth over a period of 5 days post treatment (Fig. 2C,D). For the control condition (0 V/cm), only few PI-positive cells were observed, corresponding to rare and scattered dead cells presenting damaged membrane. As expected, permeabilization increased with electric field intensity. At 400 V/cm, there were still some non-permeabilized cells in the core of the MCTS. When using the highest electric intensities, 600 and 800 V/cm, a concentric permeabilization was observed with an intense staining at the external layer of the MCTS. PI quantification showed no significant differences between 600 and 800 V/cm intensity. Nevertheless, at 800 V/cm pulsed electric field strength, a slight but not significant decrease of MCTS growth rate was observed during two days following the treatment. Based on these results, 600 V/cm was selected as an optimal condition to promote cells in-depth reversible permeabilization, without affecting cell viability.

### Irreversible cytotoxic effect of dual treatment with P-A PBS and EP on HCT 116-GFP MCTS

**Figure 3:**
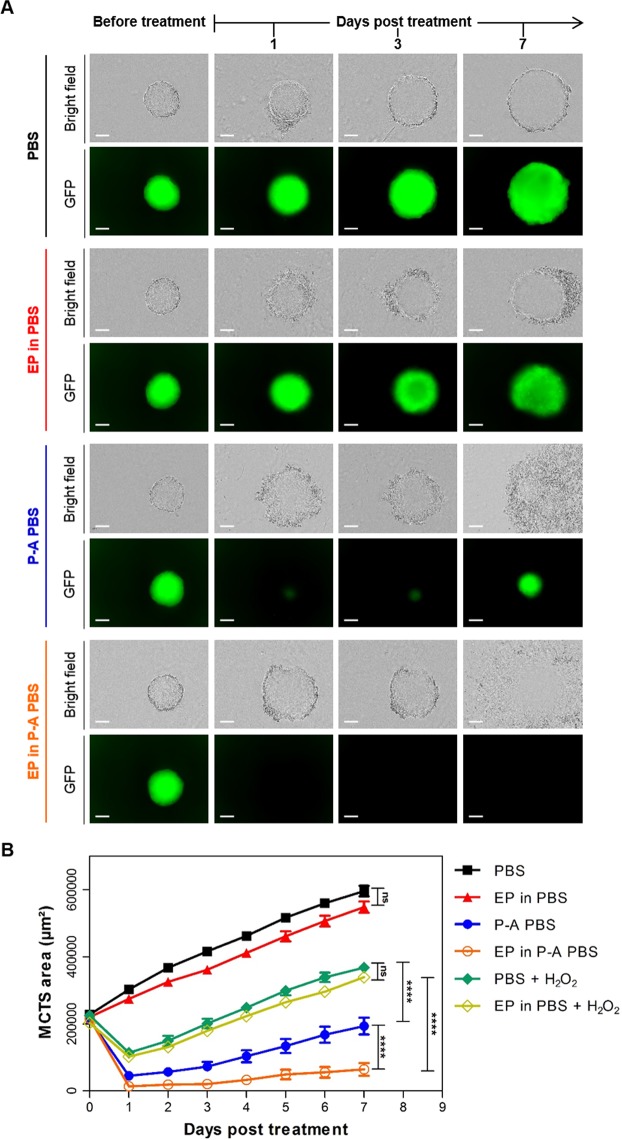
Potentiation of the P-A PBS cytotoxic effect on MCTS submitted to EP. HCT 116-GFP MCTS were put in either PBS, P-A PBS or PBS containing 680 µM of H_2_O_2_ (negative control) before being electropulsed and let for 4 hours. MCTS were then placed in culture medium for growth follow-up over 7 days post treatment. (**A**) Wide field micrographs (bright field and GFP fluorescence) before treatment (day 0) and 1, 3 and 7 days after treatment. Scale bar: 200 µm. (**B**) Growth curves representing viable MCTS (GFP fluorescence) area over time after treatment. Representative images of N = 4 experiments (n = 6 MCTS per experiment) for PBS, EP in PBS, P-A PBS and EP in P-A PBS (8/24 MCTSs regrew after dual treatment, represented by the explicit error bar occuring in the P-A PBS group) and N = 3 experiments for PBS + H_2_O_2_ and EP in PBS + H_2_O_2_ (n = 8 MCTS per experiment). Two-way ANOVA ****p < 0.0001.

**Figure 4:**
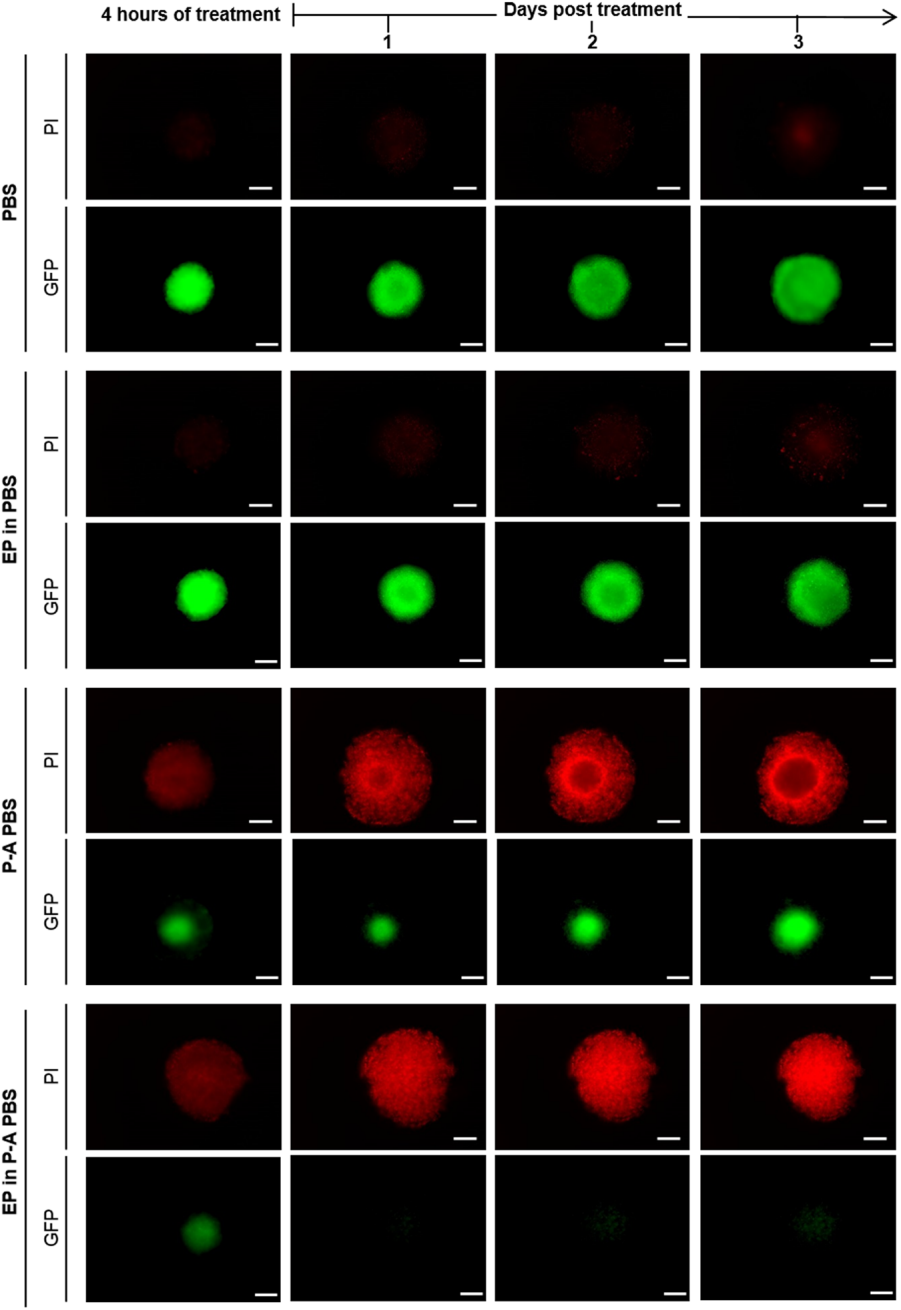
PI penetration in dead cells after MCTS dual treatment. HCT 116-GFP MCTS were incubated with P-A PBS containing 1 µM of PI during 4 hours and then placed in culture medium containing 1 µM PI for wide field fluorescence microscopy. T_0_ represents time shortly after treatment. Representative images of MCTS with Propidium iodide (PI) in red and GFP in green. Scale: 200 µm.

The macroscopic morphological changes and cell viability after dual treatment with EP (600 V/m) and 120 seconds P-A PBS were investigated by live fluorescence microscopy. Both bright field and GFP fluorescent micrographs are shown in Fig. 3A. MCTS treated with P-A PBS displayed the same morphology and growth as the one described previously in Fig. 2. When MCTS were submitted to EP in addition to P-A PBS, a morphology similar to the P-A PBS MCTS group was observed on bright field images until day 3. Seven days after treatment the 3D structure of MCTS was completely ruined. GFP fluorescence micrographs showed that entire MCTS were affected one day after the treatment. The cells lost their fluorescence within 1 day and there was no recovery even 7 days after treatment. These results were confirmed when propidium iodide was added to MCTS after treatment, showing that PI-positive cells (dead cells) within the MCTS were GFP negative (Fig. 4). Interestingly, micrographs showed that a PI-positive necrotic core began to appear within the MCTS after 8 days of culture, which means that at the time of treatment, the core included viable cells, which is in line with literature findings concerning early developmental stages of MCTSs^36^. MCTS area was measured daily and GFP fluorescence micrographs were used to obtain growth curves (Fig. 3B). A decrease of more than 95% of the mean area was observed one day after treatment for MCTS dually treated with P-A PBS and EP, whereas MCTS treated with P-A PBS alone displayed a decrease of 75%. Up to 2 days after the treatment, the differences between the two treated conditions became significant, with a p < 0.0001 after 4 days. Our results demonstrate that the cytotoxicity of P-A PBS could be greatly enhanced by means of EP. Videos available on Supplementary Data showed that complete cell death occurred between 6- and 7-hours post-treatment.

In order to assess the involvement of H_2_O_2_ in the cytotoxicity of the P-A PBS, additional experiments were performed with 680 µM of hydrogen peroxide (equal to the concentration that was quantified in Supplementary Table 1) added to the PBS medium in combination with EP. The results showed that PBS containing 680 µM H_2_O_2_ was less toxic on MCTS than P-A PBS (Fig. 3B). According to the obtained growth curves, a decrease of 49% of the mean area was observed one day after treatment for MCTS treated with PBS containing H_2_O_2,_ whereas MCTS treated with P-A PBS displayed a decrease of 75%. There was no significant difference when PEF was performed in presence of H_2_O_2_, showing that PEF potentiation of P-A PBS cytotoxicity could not be attributed to H_2_O_2_.

### Early DNA damages and caspases activation induced by dual treatment with EP and P-A PBS

**Figure 5:**
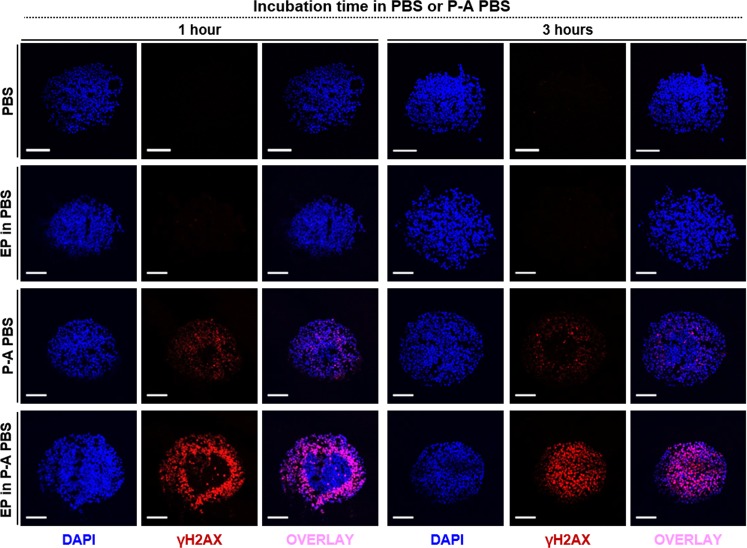
Combination of P-A PBS and EP enhanced DNA damages within MCTS. MCTS were fixed in formalin at either 1 or 3 hours post treatment (control and EP in PBS) or P-A PBS (P-A PBS and EP in P-A PBS) before cryo-freezing in OCT. Five µm-thick sections stained with DAPI (blue) and antibodies against γH2AX (Alexa 594, red) were analyzed with a confocal microscope. Scale bar = 100 µm. Representative images of N = 2 experiments, n = 10 MCTS per experiment per condition.

DNA damages were evaluated by immuno-detection of phosphorylated form of histone H2A, the γH2AX, on 5 µm-thick MCTS cryo-sections at 1 and 3 hours post treatment (Fig. 5). MCTS treated with P-A PBS displayed a similar γH2AX signal pattern 1 or 3 hours post treatment, with localized peripheral fluorescence within the MCTS. MCTS undergoing P-A PBS combined with EP showed a large number of positive γH2AX cells and a stronger signal intensity, compared to MCTS treated only with P-A PBS. One hour after treatment, the EP in P-A PBS MCTS exhibited an intense signal-emitting concentric layer of H2AX-positive zones. Within the MCTS core, there were still some cells with unaltered histones H2A. After 3 hours of treatment, the γH2AX signal became more homogenous and extended throughout the whole MCTS. The γH2AX staining patterns were clearly visible in micrographs acquired at higher magnification, a majority of whole-nuclear γH2AX signal was observed when dual-treatment was performed (Supplementary Fig. 2). Control and EP MCTS displayed few if any cells exhibiting a positive γH2AX signal 3 hours after treatment. These results show that when EP is performed in addition to P-A PBS, the DNA damages are more intense and propagate all the way through the MCTS.

**Figure 6:**
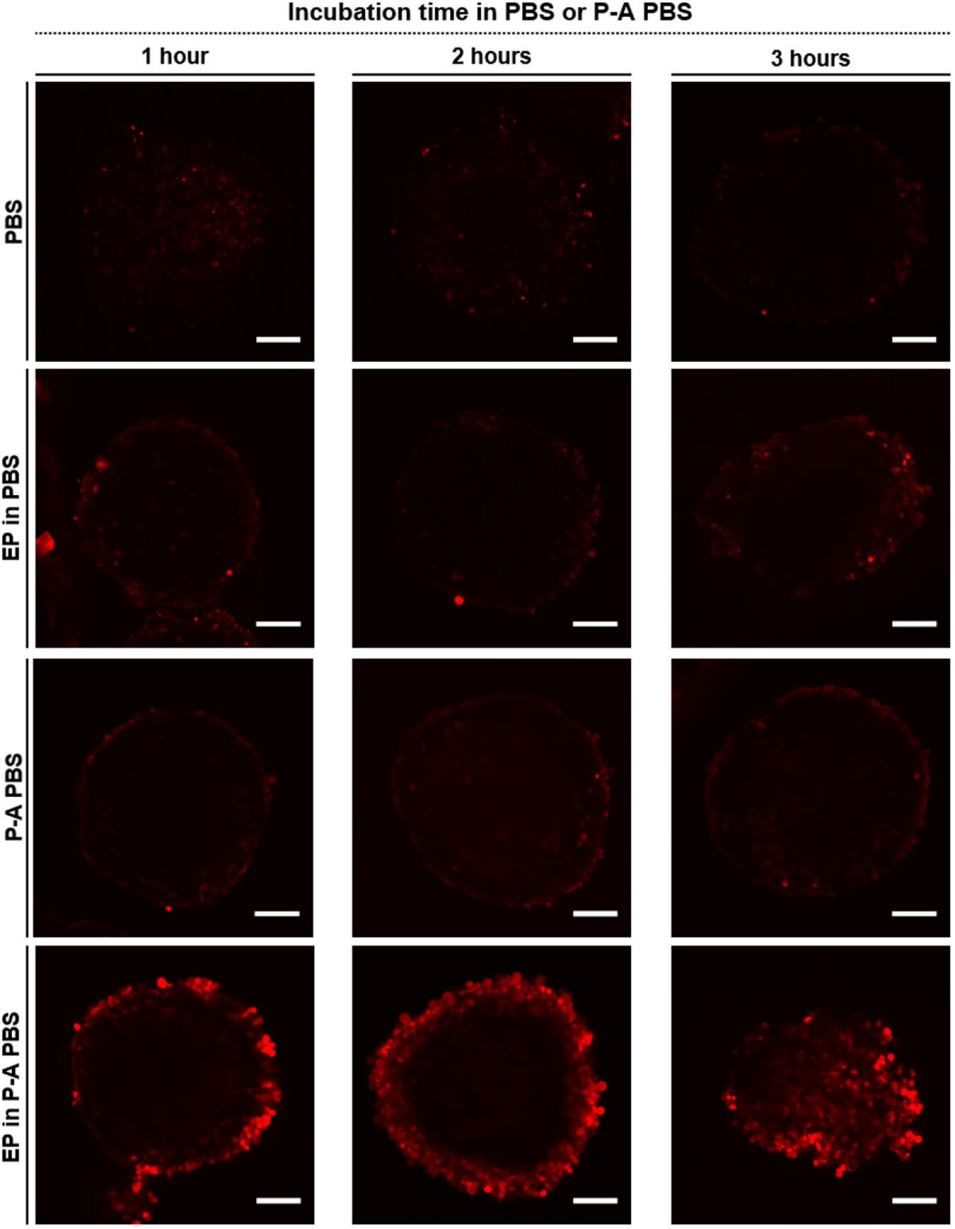
High and early activation of caspase 3/7 induced by P-A PBS and EP combination. HCT 116-GFP MCTS were incubated with FLICA reagent (caspase 3/7 dye) at different time points after treatment (1, 2 or 3 hours). Fixed whole MCTS were imaged under a confocal microscope to detect FLICA (λ = 559 nm). Scale bar: 100 µm. Representative images of N = 4 experiments, n = 4 MCTS per experiment per condition.

In further analyses, we evaluated the kinetics of apoptosis induction after treatment. The FLICA^TM^ fluorescent reagent was used to detect activated caspases 3/7 (red-fluorescent signal) at 1, 2, or 3 hours post treatment. Whole MCTS were fixed in formalin and were imaged under a confocal microscope. Micrographs shown in Fig. 6 display z-stacks taken in the middle of the MCTS. Control (PBS), EP in PBS and P-A PBS treated-MCTS did not show major caspase activation over 3 hours. In the dual treatment group, we observed a peripheral signal that was more intense than the one observed in the other groups as soon as 1 hour after treatment. Two hours after treatment, a concentric pattern of caspase activation was evident, and occupied a thicker outer layer than the one observed 1-hour post treatment. Finally, 3 hours after treatment, the MCTS displayed a distorted shape as well as a smaller volume than the MCTS in other groups, and exhibited an intense florescence signal across the entire MCTS.

Altogether, these results show that γH2AX induction and activation of Caspase 3/7 after EP treatment in the presence of PA-PBS were occurring faster than when P-A PBS treatment was applied alone. Secondly, such damages were occurring in deeper layers of the MCTS when the combined treatment was applied.

### Assessment of early ultrastructural modifications 1-hour after treatment

**Figure 7:**
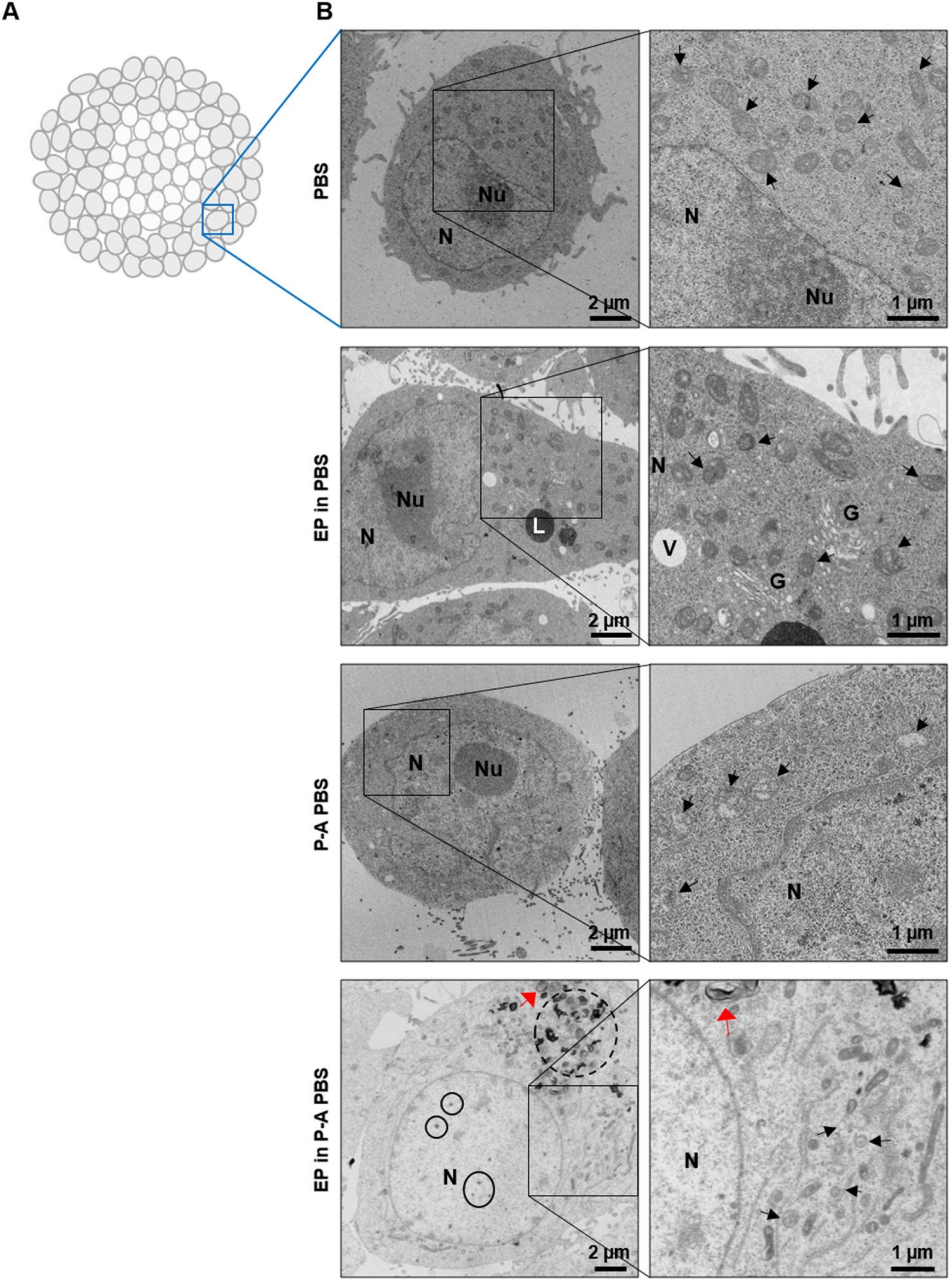
MCTS ultrastructure modifications 1-hour after treatment. (**A**) Schematic representation of the zone within the MCTS, which was imaged by transmission electron microscopy (TEM). (**B**) TEM micrographs showing representative zones in different treatment groups and at different scales. Left column showing representative micrographs of individual cells, and right column the corresponding magnified views of organelles. (N = nucleus, Nu = nucleolus, L = lysosome, V = vacuole, G = Golgi apparatus, black arrows indicate some of the mitochondria, red arrow points to lamellar bodies, autophagic vacuoles and pyknosis/karyorrhexis are surrounded with, respectively, black dashed- and solid- line circles).

The ultrastructure of MCTS cells was assessed with transmission electron microscopy (TEM) (Fig. 7). The periphery of MCTS treated with the dual treatment was characterized by an outer layer of large and moribund cells, which exhibited large and swollen nuclei with either a condensed nucleolus (pyknosis) or nuclear fragmentation (karyorrhexis) (indicated by black solid line circles in Fig. 7). These alterations, showing the destructive fragmentation of the nucleus of a dying cell, could occur as a result of either programmed cell death or necrosis. The cytosol of peripheral cells was filled with organelles debris, lysosomes, autophagosomes (indicated by black dashed line circles) and lamellar bodies (pointed with red arrows). Large autophagic vacuoles were observed in high number in dually treated specimen, even at the core of the MCTS, compared with P-A PBS-treated MCTS (black dashed line circle in Supplementary Fig. 3). The mitochondria were shrunk and their membrane was thick (indicated by black arrows). More precisely, the occurrence of inner membrane mitoptosis (IMM) was observed, where cristae and internal matrix of the mitochondria were lost while the external mitochondrial envelope remained unaltered. These structures were also observed in some cells of P-A PBS-treated MCTS, but to a smaller extent. When MCTSs were treated with P-A PBS only, cells often exhibited condensed nucleoli (pyknosis), while mitochondria exhibited thinner mitochondrial membranes and mitochondrial matrix loss, indicated by black arrows in the panels (Fig. 7). Most of the cells in the MCTS, which were exposed to the PBS alone, exhibited normal morphologies of the cytosol, nuclei, and cell organelles. When MCTSs were submitted to EP in PBS, a slightly larger amount of vacuoles and lysosomes was observed (denoted with letter V and L, respectively in Fig. 7). The membranes of mitochondria were more electron dense than the ones observed in the PBS control, and the Golgi apparatus was slightly dilated (denoted with letter G in Fig. 7). These results suggest that in addition to early apoptosis, dual treatment can also induce autophagy, which allows a methodically arranged degradation of dysfunctional cellular organelles.

## Discussion

In this study, we investigated the effect of a dual treatment with P-A PBS and EP in order to increase the cytotoxicity of the plasma-activated liquid. To test the efficiency of the dual treatment, we used a three-dimensional cancer cell model made of human colorectal cancer cells. In the first approach, we had to determine the optimal conditions for PBS exposure to a CAP jet, and to assess the most appropriate parameters for the electropermeabilization of HCT 116-GFP MCTSs. The efficacy of P-A PBS depends on its content of reactive species, which indeed depends on the operating conditions used for PBS activation (such as the gap between the plate containing PBS and the plasma jet, the gas flow rate, the plasma exposure time), and also the used plasma jet setups (configuration, working gas, power supplies, plasma temperature), which directly affect the produced RONS ratio^12^. Using MCTS as a model to predict the treatment’s response was a reliable approach making sure to take into account the growing rate of MCTS^36^. Indeed, small size MCTS display higher growing rate than larger ones, and the different organization of the layers between the center and the periphery^19,35^. Our previous studies demonstrated that plasma exposure of cell culture medium during 120 seconds allows efficient inhibition of HCT 116 human colorectal cancer cells MCTS growth^9^. Yet, cell culture medium comprises several components that might interact with plasma and might potentially generate harmful byproducts, therefore we chose to assess the effectiveness of plasma-activated PBS, which only contains salts. In order to correlate plasma activation time with the activated liquid’s efficacy, we screened several exposure times of PBS to the CAP jet and we measured the concentration of two specific RONS species (H_2_O_2_ and NO_2_^−^). Even if the levels of these RONS species are considered a good indicator for the cytotoxicity of PAL to cancer cells, the effect of P-A PBS should not be linked exclusively to the presence of RONS. Indeed, it was already shown^9^ and confirmed in our study, that the presence of H_2_O_2_ alone is not sufficient to obtain the same cytotoxic effect on cancer cells. Plasma activated liquids generally involve other species that might be toxic to cells. The latter include hydrogen peroxide and nitrite/nitrate, but also acids and other complex aqueous byproducts, which certainly simultaneously contribute to the observed cytotoxicity to cancer cells. Nevertheless, the 120 seconds exposure time led to the production of 677 µM of hydrogen peroxide and 107 µM of nitrite, respectively, and induced cell death in the outer layer of the MCTS. The level of hydrogen peroxide produced in the PBS after the 240 seconds activation protocol was indeed higher (>1000 µM), but the osmolarity of the solution increased beyond the physiological range of 300 mOsm/L, and induced moderate osmotic stress, that has severe consequences to the cell^37^. We thus chose to perform a 120 seconds CAP jet activation, and combine the use of P-A PBS with EP to test the efficiency of the dual treatment, and enhance P-A PBS cytotoxicity within the MCTS. In order to achieve high, efficient and reversible cell permeabilization within the MCTS, the ESOPE protocol (eight pulses lasting 100 µs at a frequency of 1 Hz)^21^ was applied. As the permeabilization of the cell membrane depends on the exposure conditions and the cell type^16^, we had to establish the electric field intensity, at which the permeabilization and the viability are the highest, and for HCT 116-GFP cells in MCTS, the fixed field intensity was 600 V/cm.

As emphasized in the results section, the P-A PBS treatment induces a transient cytotoxic effect, which is not strong enough to affect the deeper layers of MCTS. Conversely, the dual treatment induces an irreversible and global effect, which sets on in the periphery, and over time propagates throughout the MCTS, and finally yields to a complete growth arrest. The immunostaining of histone H2AX allowed us to evidence DNA damages occurring within the MCTS at different post-exposure time points. Besides, additional tests evidenced that the areas displaying DNA damages also presented an earlier and more intensive activation of caspases 3/7, indicating that the dual treatment leads to an apoptotic cell death in a higher number of cells. Both, the γH2AX induction and the activation of Caspase 3/7 occurring after EP treatment in the presence of PA-PBS set on more efficiently in comparison to what we observed after P-A PBS treatment alone. Taken together, our results thus substantiate that electropermeabilization enhances the cytotoxic effects of plasma activated PBS, which, over time, propagate from MCTS periphery into the core. These global damages result in the ablation of most if not all cells that constitute MCTS.

Previous studies showed that MCTS were able to reorganize and decrease their quiescent cells proportion to switch to a more proliferative behavior under pulsed electric field^36^. We thus assume, it might be possible MTCS respond in the same manner to the P-A PBS treatment. This would explain the growth pattern of P-A PBS-treated MCTS. After an initial decrease of MCTS growth, from day 2 onward, this group exhibited the same growth speed (same growth curve slope) as the controls, indicating that the core of the MCTS continued to grow.

As propidium iodide was taken up by the cells in all layers of MCTS, we first assumed that we enhanced the cytotoxicity of P-A PBS by means of EP-induced cell permeabilization, which allowed a better RONS penetration within the MCTS. Moreover, the efficiency of P-A PBS probably strongly depends on the quantity of produced hydrogen peroxide and nitrite species^38^. As, to our knowledge, there were no published studies evaluating the toxicity of H_2_O_2_ after application of pulsed electric field to MCTS, we also considered potential cellular effects of H_2_O_2._ In order to assess a potential toxicity of hydrogen peroxide, we added synthetic H_2_O_2_ to MCTS and we showed that EP did not potentiate the cytotoxic effect of H_2_O_2,_ as there was no significant difference in MCTS growth when EP was performed in presence of H_2_O_2_ in PBS. Therefore, the enhancement of PA-PBS toxicity by EP treatment can be attributed either to the presence of other species (NO_2_^−^, NO_3_^−^, …) in plasma-exposed liquids or, to H_2_O_2_ and NO_2_^−^, which act in synergy to kill cancer cells, as described by Girard et co-workers in the 2D cell model^38^. Thus, potentiation of P-A PBS cytotoxicity by EP might be effective only when the two species are present.

In order to assess the morphological changes occurring rapidly in MCTS on cellular ultrastructural level, we performed the transmission electron microscopy (TEM). The structural anomalies were observed in the nuclei and mitochondria in the P-A PBS group, while the dual treatment affected all cellular components. The condensation of chromatin in cellular nuclei (also known as pyknosis) and the fragmentation of the nucleus (karyorrhexis), which were observed in P-A PBS group and EP in P-A PBS group, respectively, correlate with the DNA damages and the onset of apoptosis^39^. The latter were confirmed by functional biochemical tests. Indeed, these nuclear structures were observed in a larger proportion when combined treatment was applied, in comparison to the unimodal P-A PBS treatment. Moreover, when compared with P-A PBS-treated MCTS, pyknosis and karyorrhexis were disseminated throughout a thicker rim in MCTS in the EP in P-A PBS-treated MCTS. Interestingly, the mitochondria shrank and exhibited matrix loss. In addition, the mitochondria were characterized by ruptures of cristae, a phenomenon that is characteristic of the inner membrane mitoptosis^40^. This kind of ultrastructure modification occurs during both apoptosis and autophagy pathway^40^. More recently, mitochondrial structural changes, such as the one observed in our investigations, were correlated to the loss of mitochondrial potential^41^. In both P-A PBS and EP in P-A PBS treated-conditions autophagic vacuoles were observed, yet, the EP in P-A PBS group exhibited the highest proportion of autophagosomes. The appearance of autophagy has been related to different kinds of cellular stress, such as nutrient starvation or oxidative stress, that lead to RONS generation^42^. Under our conditions, MCTS were kept in PBS for four hours after treatment, which might have induced nutrient starvation, but observed control MCTS’s cells rarely exhibited autophagic vacuoles and intracellular vesicles. While autophagic vacuoles were not present in the EP in PBS group, the cells did contain a large fraction of intracellular vesicles. Nevertheless, the vesicles in the EP-treatment group did not contain any organelles debris, and probably corresponded to vesicles that are generated due to the increased micropinocytosis and endocytosis, which occur after cellular exposure to pulsed electric fields^43,44^. As autophagosomes were mainly present in P-A PBS and EP in P-A PBS treated-conditions, we can conclude that in our case such intracellular structures are specific to the plasma-activated liquids treatment.

Steuer *et al*. hypothesized that pulsed electric fields could enhance treatment efficacy by altering cell-cell junctions of adjacent cells^45,46^. These cell-cell junctions’ alterations have also been reported for endothelial cells *in vitro* and *in vivo*^47^. Indeed, EP leads to an osmotic swelling of the cells and a reorganization of the cytoskeleton, leading to a transient increase of the intercellular space^48^. These alterations are reversible in absence of cytotoxic drugs^49^. In parallel, Funk and Krise showed that hydrogen peroxide induced reductions in lateral membrane diffusion, which might be responsible for substrates’ accumulation within cells^50^. This supports the finding that the application of both techniques has an increased and irreversible cytotoxic effect, which can be attributed to the cumulative effects of EP and RONS. Based on this insight, we suggest that the combination of P-A PBS with EP may lead to both, higher plasma-activated liquid penetration, due to induced membrane permeabilization, and an alteration of the cell-cell junctions within the MCTS. The loss of cellular cohesion hence additionally promotes the penetration of RONS in the deeper layers of MCTS, allowing cell death within the core of the MCTS, resulting in an irreversible cytotoxic effect.

## Conclusion

In the present study, we demonstrated that electropermeabilization greatly enhances plasma-activated PBS cytotoxic effect in a three-dimensional human colorectal cancer cell model. The *in vitro* approaches developed here may open up new possibilities for further investigations of the anti-cancer potential of pulsed electric fields in combination with plasma-activated PBS, including the ones that could be applied *in vivo*.

## Methods

### Cell Culture and MCTS generation

Green fluorescent protein (GFP) transduced^51^ human colorectal carcinoma cells HCT 116, supplied by ATCC^®^ CCL-247^TM^ were cultured in Dulbecco’s Modified Eagle Medium (DMEM, Gibco-Invitrogen, Carlsbad, USA) containing 4.5 g/L glucose, L-Glutamine and pyruvate, supplemented with 1% of penicillin/streptomycin and 10% of fetal bovine serum. The cells were cultured in a controlled humidified atmosphere (5% CO_2_, 37 °C). HCT 116-GFP MCTS were made using the non-adherent technique in Costar® Corning*® Ultra-low attachment* 96-well plates (Fisher Scientific, Illkirch, France). Five hundred cells were seeded per well in 200 µL of cell culture medium, and plates were cultivated in 5% CO_2_ humidified atmosphere at 37 °C. Single MCTS were obtained in each well 24 hours after seeding. At day 5 after seeding the MCTS reached 450–500 µm of diameter and were considered ready for treatment.

### MCTS treatments

#### Plasma-activated PBS (P-A PBS)

The low temperature plasma jet was generated in ambient air by using dielectric barrier discharge as described elsewhere^10^. In summary, the plasma jet was produced with a gap distance of 10 mm between two aluminum tape electrodes wrapped around a quartz tube. The flow rate of helium gas was 3 L/min. A mono-polar square pulse with 10 kV of voltage magnitude, 10 kHz of frequency and 1 μs duration ignited the electric discharge. For PBS activation, 100 µL of PBS (Eurobio’s phosphate buffered saline modified without Ca^2+^ and Mg^2+^, Sigma) was exposed to the cold atmospheric plasma jet in the wells of 96-well round adherent bottom plates for 60, 120 or 240 seconds. The distance between the liquid surface and the plasma jet tube output was fixed at 2 cm, based on our previous work^9^. Hydrogen peroxide, nitrite and nitrate content generated in P-A PBS were characterized using Fluorimetric Hydrogen Peroxidase and Nitrite/Nitrate colorimetric assay kits (Sigma Aldrich) according to manufacturer’s instructions. The osmolarity of P-A PBS was measured with a cryoscopic osmometer, OSMOMAT 030 (Gonotec) within a maximum of 30 minutes after plasma exposure. For the treatment, MCTS were incubated in 80 µL of P-A PBS (within 30 minutes after plasma jet exposure) for 4 hours, and MCTS were incubated in either PBS or PBS containing 680 µM of hydrogen peroxide (FOURNISSEUR) served as the controls. After the 4 hours treatment incubation, treated media were removed and fresh culture medium was added.

#### Electropermeabilization

MCTS electropermeabilization was performed with a unipolar square-wave electric pulses generator ELECTROCELL S20 (βtech, Toulouse, France) with a high-voltage maximum of 1.5 kV connected to flat parallel stainless steel electrodes with the inter-electrode distance of 4 mm. Electric parameters corresponded to electrochemotherapy ESOPE protocols^21^. Eight pulses lasting 100 µs at a frequency of 1 Hz were applied at optimized (600 V/cm) electric field intensity and at room temperature. Electropermeabilization was also performed in PBS containing 680 µM hydrogen peroxide as a control experiment. MCTS were placed either in a 100 µL liquid drop of PBS, P-A PBS or PBS + H_2_O_2_ (dual treatment) and they remained in the liquid up to 1 minute prior electropermeabilization.

**Figure 8:**
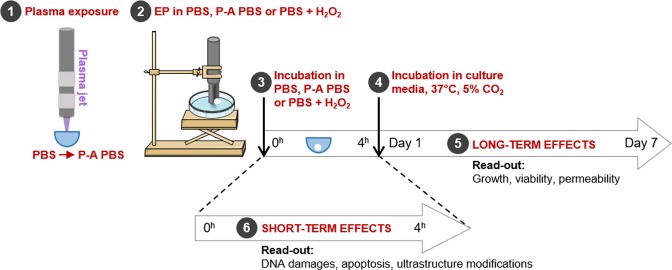
Schematic view of the protocol for HCT 116-GFP MCTS treatment and data analysis. (**1**) PBS was exposed to the plasma jet. (**2**) MCTS were placed in a 100 µL liquid drop of either PBS (**EP in PBS**), P-A PBS (**EP in P-A PBS**) or PBS + H_2_O_2_ (**EP in PBS** **+** **H**_**2**_**O**_**2**_) up to 1 minute prior application of EP. (**3**) MCTS were then kept in the treatment media for 4 hours of incubation at 37 °C, 5% CO_2_. Three other groups were not submitted to EP and were just incubated in the **PBS**, **P-A PBS or PBS** **+** **H**_**2**_**O**_**2**_. (**4**, **5**) MCTS were rinsed and placed in the fresh culture medium and placed at 37 °C, 5% CO_2_ for MCTS growth follow-up during 7 days. DNA damages, apoptosis and ultrastructure modifications were assessed after the incubation period of 1, 2, 3 and 4 hours, and were assessed either on living cells or on fixed MCTS, according to the described protocol (**6**).

Figure 8 illustrates the treatment protocol applied in this work and schematizes the materials and methods section.

### MCTS analysis

#### Growth of MCTS and analysis of micrographs with ImageJ

After 4 hours of immersion in PBS or P-A PBS, MCTS were rinsed twice with PBS and placed in cell culture medium, where MCTS growth and viability was continuously followed during 7 days (4 days after treatment, fresh culture medium was added to the MCTS) using IncuCyte Live Cell Analysis System Microscope with magnification x10 objective. Micrographs were exported from the IncuCyte software and the GFP green fluorescence was used to set a threshold mask on MCTS and to measure the area with the ImageJ software.

#### Cell membrane permeabilization upon EP treatment

Propidium iodide (PI) was used to detect cell membrane permeabilization after MCTS electropermeabilization at different electric field intensities. 100 µM of PI in PBS was used in the electropermeabilization experiment, where MCTS were placed in a 100 µL drop of the PBS containing PI, which was placed between the electrodes and the electric field pulses were applied. Five minutes after the MCTS were rinsed and the PI fluorescence (λ_exc_ = 535 nm, λ_em_ = 617 nm) was monitored on live MCTS under a wide field Zeiss Primovert microscope (Carl Zeiss, Marly Le Roy, France), equipped with a DMIRB CoolSnapFx HQ2 camera. Images were then analyzed with the ImageJ software.

#### Cell viability

Propidium iodide was also used to evaluate cells viability after treatment (when membrane permeabilization occurred due to cell death). MCTS were electropulsed in either PBS or P-A PBS, and kept for 4 hours in newly prepared PBS or P-A PBS solution containing 1 µM concentration of PI. After the 4 hours incubation, MCTS were rinsed twice with PBS and placed in fresh cell culture media, also containing 1 µM of PI. PI penetration to dead cells within MCTS was continuously followed during 3 days with IncuCyte Live Cell Analysis System Microscope.

#### Transmission electronic microscopy

TEM was used to observe the cellular ultrastructure modifications in MCTS before and after the physical treatments. Treated (P-A PBS, EP in P-A PBS) and control (PBS, EP in PBS) MCTS were retrieved 1 hour after treatment, fixed with 2% glutaraldehyde in 0.1 M sodium cacodylate buffer, post-fixed with 1% osmium tetroxide, gradually dehydrated in ethanol, and embedded in the Embed 812 resin (Electron Microscopy Sciences) using a Leica EM AMW automated microwave tissue processor for electron microscopy. Stained 70-nm sections (3% uranyl acetate in 50% ethanol and Reynold’s lead citrate) were observed with a HT 7700 Hitachi transmission electron microscope, operating at an accelerating voltage of 80 kV and equipped with a CCD AMT XR41 camera.

#### Caspases 3/7 activity detection in MCTS

Image-iT live red caspase −3 and −7 detection kit for microscopy (Invitrogen) was used according to the manufacturer’s instructions to detect caspase activation at different time-points. Briefly, HCT 116-GFP MCTS were treated as described in section 2, where MCTS were treated and sequentially incubated (following 1, 2 or 3 hours after treatment) with 60 µL of FLICA reagent for 45 minutes at 37 °C, 5% CO_2_. MCTS were then rinsed twice with PBS before being fixed in 60 µL of kit fixative solution for 24 hours at 4 °C. Fixed whole MCTS were imaged under FV1000 confocal microscope (Olympus, France) at a magnification x20. The emitted light from FLICA reagent was collected through a 610–650 nm band pass filter (Texas red).

#### Immunofluorescence on MCTS cryo-sections

Treated (P-A PBS, EP in P-A PBS) and control (PBS, EP in PBS) were retrieved after 1, 2 or 3 hours of treatment and fixed using neutral-buffered formalin (Sigma) for 2 hours at room temperature. MCTS were then placed in PBS-sucrose 15% and 30% sequentially for 12 hours at 4 °C. Shandon Cryomatrix™ resin (Thermofisher Scientific) was used to embed MCTS and cryo-freezing was performed using isopentane and liquid nitrogen. Blocks were sliced in 5 μm-thick sections and mounted on microscopy slides. Slides were blocked with PBS-1% BSA-0.5% Triton X-100 and stained with primary antibodies against phosphorylated Histone H2AX^52^ (rabbit monoclonal, Cell Signaling Technology) at 1/500 dilution for 1 hour at room temperature. Goat anti-rabbit IgG coupled with Alexa Fluor 594 (Life Technologies) at 1/800 dilution were used as secondary antibodies for 30 minutes staining at room temperature. Slides were mounted in Vectashield mounting medium with DAPI (Vector Laboratories) and imaged with a FV1000 confocal microscope (Olympus, France) at different magnifications (x20 and x40).

### Statistical analysis

Data are given as mean ± (Standard Error of the Mean) SEM with *N* replicates and *n* denoting the number of MCTS in each replicate. Overall statistical significance was set at p < 0.05. Differences between values were assessed by one or two-way ANOVA and curve slopes were obtained by linear regression analysis.

## Supplementary information

Supplementary data

Video A

Video B

Video C

Video D
