# Supplementary material for: Pulsed Electric Field Treatment Enhances the Cytotoxicity of Plasma-Activated Liquids in a Three-Dimensional Human Colorectal Cancer Cell Model
Source: Sci Rep. 2019 May 20;9:7583. doi: 10.1038/s41598-019-44087-5 (PMC6527570; doi:10.1038/s41598-019-44087-5)
Supplement: Supplementary file 1 — Supplementary data [file 41598_2019_44087_MOESM1_ESM.docx]

**Title: Pulsed Electric Field Treatment Enhanced the Cytotoxicity of Plasma-Activated Liquids on a Three-Dimensional Human Colorectal Cancer Cell Model.**

**Authors:** Elena Griseti*^1, 2^*, Jelena Kolosnjaj-Tabi^1^, Laure Gibot*^1^*, Isabelle Fourquaux^3^, Marie-Pierre Rols*^1,^* Mohammed Yousfi^2^, Nofel Merbahi*^2^** & Muriel Golzio*^1^**.

**SUPPLEMENTARY INFORMATION**

**Videos:**

See attached files.

**HCT 116-GFP MCTS morphological changes after dual treatment with P-A PBS and EP.** Bright field and GFP micrographs were acquired with the IncuCyte Live Cell Analysis System Microscope hourly during 7 days. The first 4 hours displayed on the videos correspond to hours of incubation either in PBS (untreated groups) or in P-A PBS (treated groups). Videos are displayed as overlays of bright field and GFP channels. (A) Untreated MCTS, bath in PBS for 4 hours; (B) EP in PBS; (C) P-A PBS; (D) EP in P-A PBS. After the 4 hours of incubation in either PBS (A, B) or P-A PBS (C, D), MCTS were placed in culture medium and put in the microscope.

**Figures:**

**Supplementary figure 1: MCTS growth follow-up after incubation in PBS or helium-exposed PBS, compared to untreated MCTS.** In order to assess the effect of PBS bath and helium gas exposition on MCTS growth, HCT 116-GFP MCTS were incubated 4 hours with these two-buffered solutions. MCTS were observed after treatment under IncuCyte Live Cell Analysis System Microscope. GFP fluorescence area was measured over time during 7 days. Area was compared with MCTS cultured in DMEM (untreated). N= 2 experiments, n=8 MCTS per conditions, mean ± standard deviation.

**
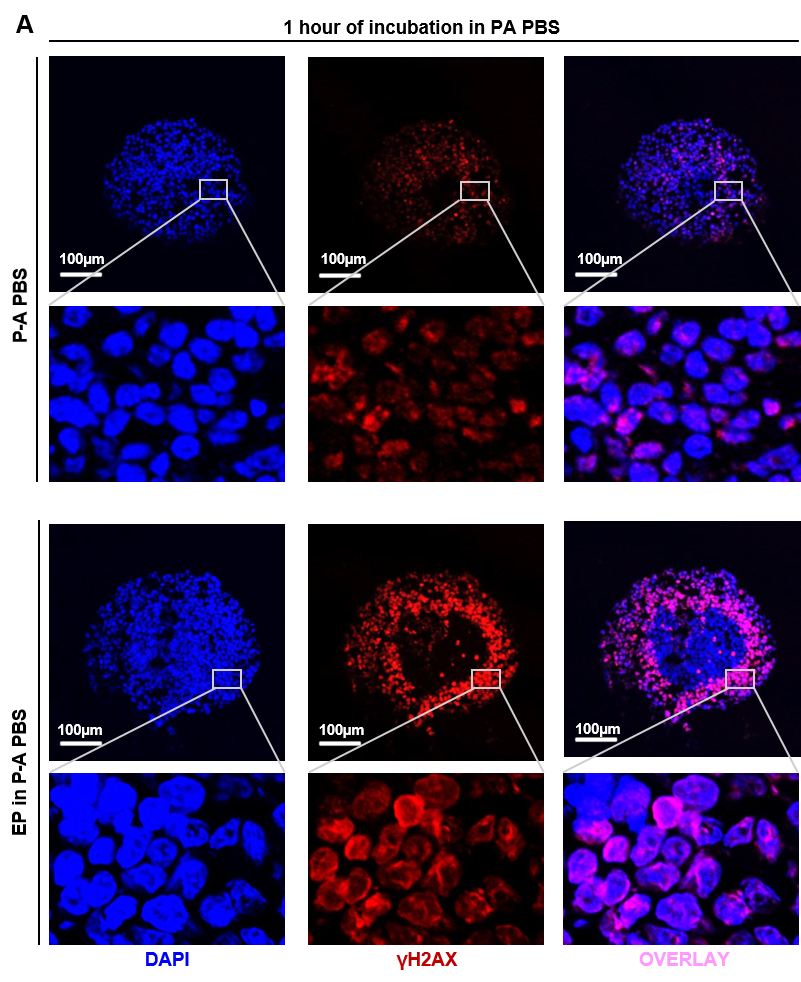

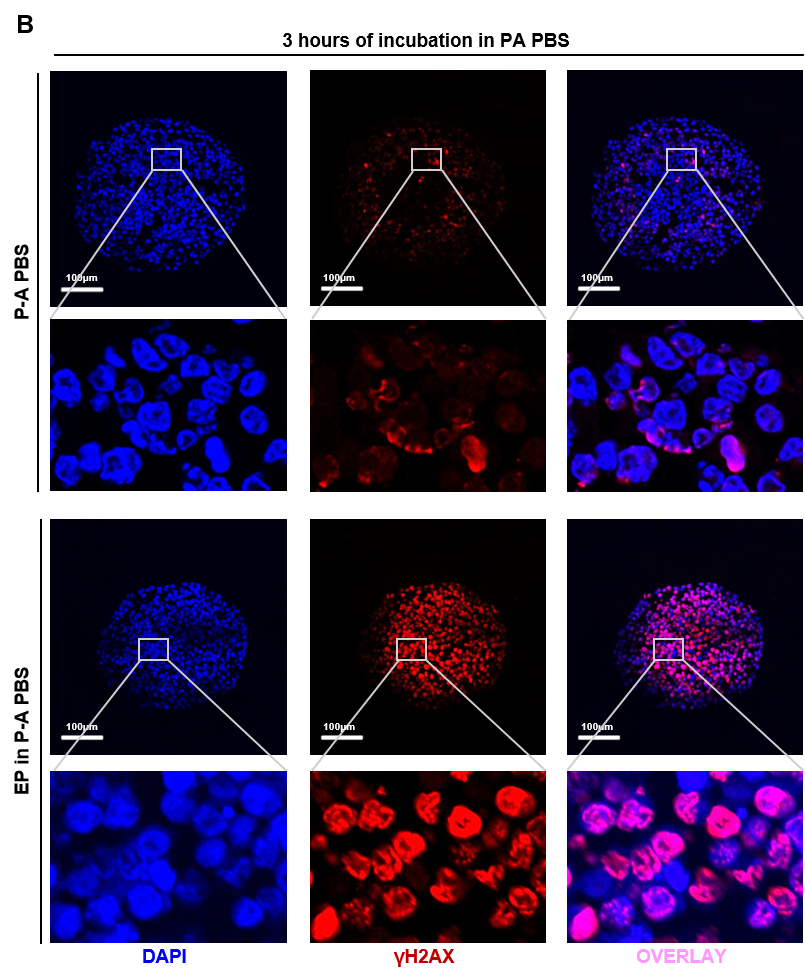
**

**Supplementary figure 2: γH_2_AX staining patterns observed within MCTS submitted to P-A PBS treatment or dual treatment with EP.** MCTS were fixed in formalin at either 1 (A) or 3 hours (B) post electropermeabilization in P-A PBS before cryo-freezing in OCT. Five µm-thick sections stained with DAPI (blue) and antibodies against γH2AX (Alexa 594, red) were analyzed with a confocal microscope at x20 and x40 magnification. Representative images of N= 2 experiments, n= 10 MCTS per experiment per condition.


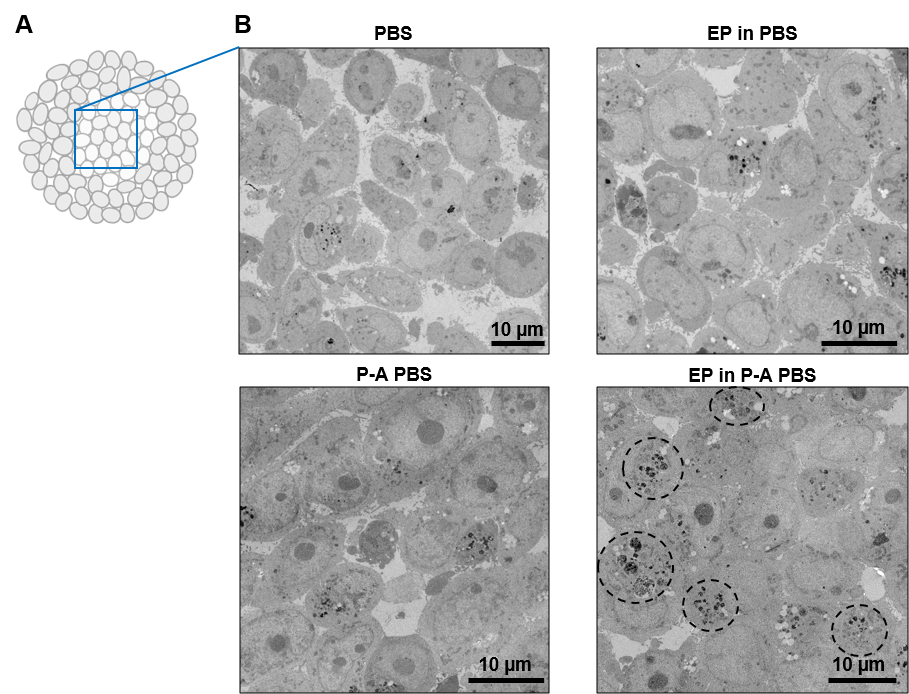


**Supplementary figure 3: Core MCTS ultrastructure modifications 1-hour after treatment.** (A) Schematic representation of the zone within the MCTS, which was imaged by transmission electron microscopy (TEM). (B) TEM micrographs showing representative zones imaged within the MCTS core in different treatment groups. Autophagic vacuoles are surrounded with black dotted circle.

**Table:**

| Plasma exposure time (s) | 0 | 60 | 120 | 240 |
| --- | --- | --- | --- | --- |
| Osmolarity (mOsmol/L) | 299.3 ± 2.1 | 330 ± 5.1 | 362 ± 5.1 | 533 ± 33.15 |
| [H_2_O_2_] (µM) | 0 | 367.5 ± 34.1 | 677.3 ± 28.5 | 1390 ± 38.6 |
| [NO_2_^-^] (µM) | 0 | 54.1 ± 6.1 | 106.7 ± 19.3 | 181.3 ± 37.3 |

**Supplementary Table 1: Osmolarity and RONS analysis of PBS activated during different plasma exposure times.** Hydrogen peroxide and nitrite content were characterized using Fluorimetric Hydrogen Peroxidase and Nitrite colorimetric assay kits. Osmolarity changes were measured with a cryoscopic osmometer, OSMOMAT. Measurements were performed on PBS within 30 minutes after its exposure to plasma jet. N= 4 experiments and n=3. Mean ± standard error mean.
